# Machine learning-based identification of heart failure with reduced ejection fraction using routine laboratory indicators

**DOI:** 10.3389/fcvm.2026.1856570

**Published:** 2026-08-10

**Authors:** Zhiping Meng, Xuezhan Qin, Binbin Liang, Wenpei Lin, Wentan Xie, Guinian Du

**Affiliations:** 1 Department of Laboratory Medicine, Guigang Clinical Medical Research Center for Medical Laboratory, Eighth Affiliated Hospital of Guangxi Medical University, Guigang City People’s Hospital, Guigang, Guangxi, China; 2 Department of Information Technology, The People Hospital of Guigang, Guigang, Guangxi, China; 3 Department of Cardiovascular Medicine, The People Hospital of Guigang, Guigang, Guangxi, China

**Keywords:** heart failure with reduced ejection fraction, routine laboratory indicators, machine learning, random forest, SHAP, clinical prediction model

## Abstract

**Background:**

Early recognition of heart failure with reduced ejection fraction (HFrEF) is clinically important because treatment pathways and prognosis differ across left ventricular ejection fraction phenotypes. When echocardiography is delayed, routinely available laboratory data may help prioritize patients for definitive imaging.

**Methods:**

This single-center retrospective study included 1,480 hospitalized patients with chronic heart failure, comprising 377 with HFrEF and 1,103 with HFmrEF/HFpEF. Baseline laboratory indicators were obtained from the first blood draw within 24 h of admission, and matched echocardiography was typically performed within 48 h. After a stratified 7:3 train-test split, preprocessing, imputation, standardization, feature selection, and model training were conducted within a leakage-free machine-learning pipeline. Cross-validated recursive feature elimination retained 13 routine indicators: proBNP, HCT, TBil, DBil, BUN, *β*2-MG, CRP, UA, Glb, GGT, MCH, LDL-C, and FIB. Six algorithms were evaluated using discrimination, calibration, decision-curve analysis, threshold analysis, and SHAP interpretation.

**Results:**

The HFrEF group showed higher proBNP, BUN, TBil, DBil, GGT, HCT, and UA levels than the HFmrEF/HFpEF group. In the independent test set, random forest and XGBoost achieved the highest AUCs (both 0.789), whereas logistic regression showed comparable discrimination (AUC: 0.784) and strong calibration (intercept 0.017; slope 1.069). Random forest had the lowest Brier score (0.152), but pairwise bootstrap comparisons showed no statistically significant AUC differences among models. At the default threshold, sensitivity was limited; however, moving the random forest threshold to 0.15 increased sensitivity to 0.912 and negative predictive value to 0.935, supporting a rule-out triage strategy for prioritized echocardiography. SHAP analysis identified proBNP as the dominant predictor, followed by HCT, DBil, Glb, UA, GGT, TBil, and LDL-C.

**Conclusions:**

A 13-indicator routine laboratory model provided moderate discrimination for distinguishing HFrEF from HFmrEF/HFpEF. Random forest had the numerically most favorable balance of discrimination and calibration, but its clinical usefulness depends on threshold optimization rather than default classification. The model should be regarded as an adjunctive triage tool pending external and prospective validation, not as a substitute for echocardiographic phenotyping.

## 1 Introduction

Heart failure (HF) is a clinical syndrome caused by structural or functional cardiac abnormalities and is characterized by compatible symptoms and/or signs together with elevated natriuretic peptide concentrations and/or objective evidence of pulmonary or systemic congestion (1). On the basis of left ventricular ejection fraction (LVEF), HF is commonly categorized as heart failure with reduced ejection fraction (HFrEF, LVEF ≤ 40%), heart failure with mildly reduced ejection fraction (HFmrEF, 41%–49%), heart failure with preserved ejection fraction (HFpEF, ≥50%), and heart failure with improved ejection fraction (HFimpEF, baseline LVEF ≤ 40% followed by a meaningful increase to >40%) (1). This phenotypic classification is clinically important because it reflects systolic function, pathophysiological heterogeneity, therapeutic decision-making, and prognosis.

Guidelines and focused updates increasingly emphasize early initiation and continued optimization of foundational pharmacotherapies for HFrEF, including ARNI/ACEI/ARB, beta-blockers, mineralocorticoid receptor antagonists, and sodium-glucose cotransporter-2 inhibitors, to reduce mortality and rehospitalization (2, 3). By contrast, the evidence base for HFmrEF and HFpEF remains more heterogeneous; available reviews and meta-analyses suggest that HFmrEF has features and treatment responsiveness intermediate between HFrEF and HFpEF, whereas sodium-glucose cotransporter-2 inhibitors have the most consistent evidence across the HFmrEF/HFpEF spectrum (4, 5).

Echocardiography remains the key modality for assessing LVEF and completing HF phenotypic classification, but it is not always immediately available in first-contact, emergency, primary-care, or resource-constrained settings. Natriuretic peptides, particularly NT-proBNP/proBNP, are central biomarkers for HF diagnosis and risk stratification, yet their discriminative value in HFmrEF/HFpEF can be modified by obesity, kidney function, functional capacity, and clinical context (6–8). Machine-learning methods have increasingly been used for HF mortality and readmission prediction, HFpEF phenotyping, treatment-response assessment, and outcome prediction (9–12). Because HF involves multisystem pathophysiology, including myocardial stress, cardiohepatic congestion, renal dysfunction, inflammation, hematologic abnormalities, and altered metabolic status, routine laboratory indicators may provide scalable phenotypic information beyond any single biomarker. We therefore developed and internally evaluated a routine laboratory-based machine-learning model to distinguish HFrEF from HFmrEF/HFpEF and to assess its discrimination, calibration, threshold behavior, interpretability, and potential use as an adjunctive triage tool.

## 2 Materials and methods

### 2.1 Study design and study population

This single-center retrospective observational study used the electronic medical record and laboratory information systems of Guigang City People's Hospital and included hospitalized patients with chronic HF from January 2019 to December 2022. Eligible patients were aged 18 years or older, had a confirmed diagnosis of chronic HF, had at least one documented LVEF value in the first echocardiographic report during hospitalization, and had the required baseline laboratory tests performed within 24 h of hospital admission. Echocardiographic examinations matched to these laboratory results were typically performed within 48 h of admission to reflect baseline status before substantial therapeutic intervention, such as extensive diuresis. The available database recorded the clinically reported LVEF value; measurement-method heterogeneity could not be fully reconstructed retrospectively and is therefore acknowledged as a limitation.

**Figure 1:**
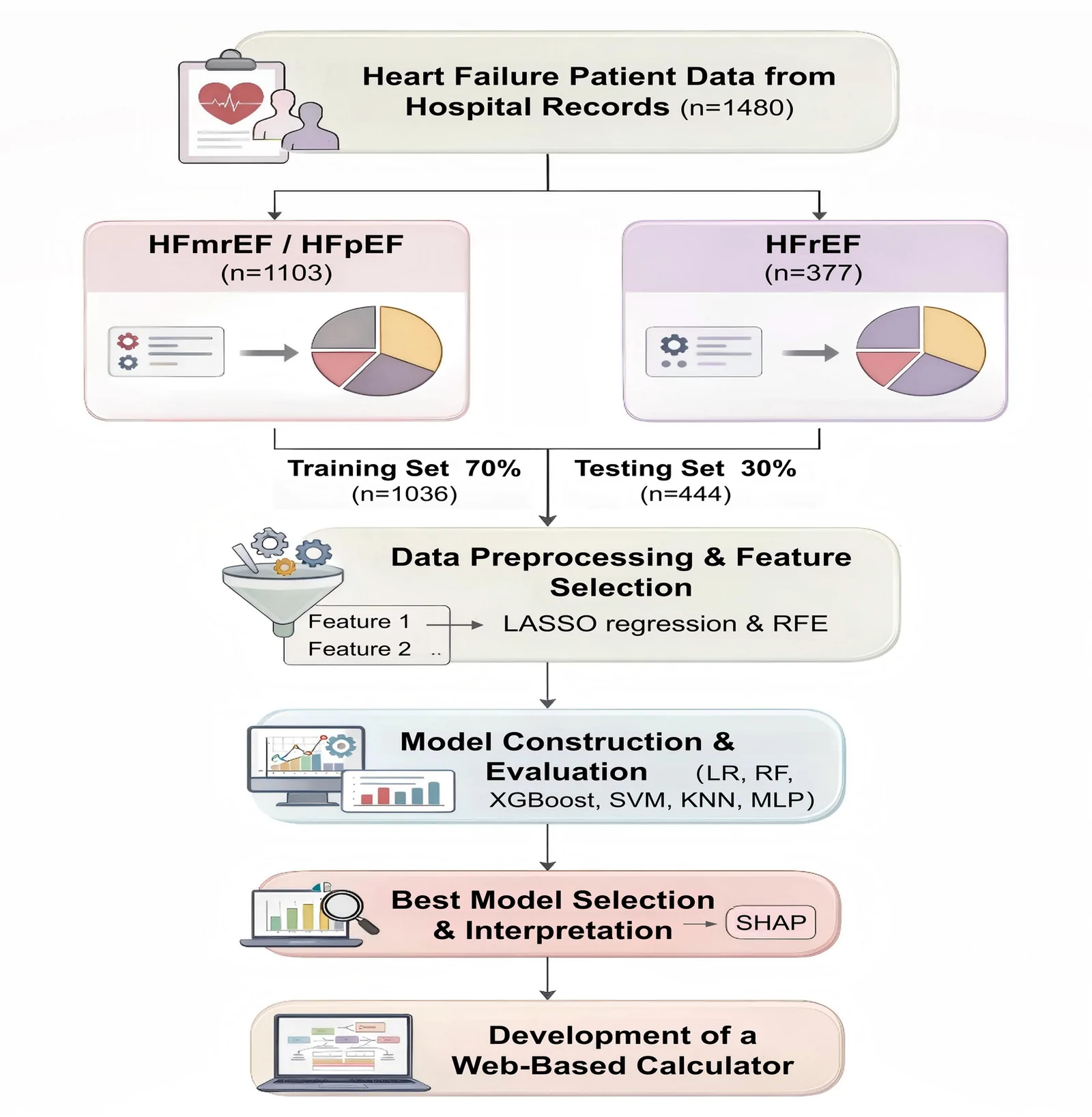
Study flowchart for patient screening, data preprocessing, model development, model evaluation, interpretation, and deployment. The workflow shows patient screening based on inclusion/exclusion criteria, baseline laboratory extraction within 24 h of admission, leakage-free preprocessing after data splitting, RFECV feature selection, development of six machine-learning models (LR, RF, XGB, MLP, SVM, and KNN), model evaluation using discrimination, calibration, threshold analysis, and decision-curve analysis, SHAP interpretation, and research-use web deployment. HFrEF, heart failure with reduced ejection fraction; RFECV, recursive feature elimination with cross-validation; SHAP, SHapley Additive exPlanations.

Patients with acute myocardial infarction, myocarditis, cirrhosis, renal replacement therapy, malignant tumors, or substantial missing data were excluded. The binary outcome was HFrEF (status = 1; LVEF ≤ 40%) vs. HFmrEF/HFpEF (status = 0; LVEF > 40%). HFimpEF could not be separately defined because prior LVEF trajectories and complete treatment histories were not consistently available in the retrospective database. The study was conducted in accordance with the Declaration of Helsinki and approved by the Ethics Committee of Guigang City People's Hospital (approval number: ELW-2025-022-01). Reporting was structured according to the TRIPOD + AI statement (13, 14). The study selection and analysis workflow are shown in Figure 1.

### 2.2 Variable collection and data preprocessing

Candidate predictors comprised routinely available laboratory indices extracted from the hospital information system, including natriuretic peptide markers, hematologic indices, renal and liver function parameters, electrolytes, inflammatory markers, coagulation variables, and lipid profiles. Raw string values containing nonnumeric symbols, such as “>”, “<”, and “%”, were cleaned and converted to numerical values where appropriate.

To rigorously prevent data leakage, data splitting was performed before preprocessing. The cohort was randomly divided into training (70%) and test (30%) sets by stratified sampling. A scikit-learn pipeline was then constructed in which missing continuous variables were imputed using k-nearest neighbors (*k* = 5), categorical variables were imputed using mode imputation, and features were standardized for scale-sensitive models. These preprocessors were fitted exclusively on the training set and then applied to the test set. Variables with more than 30% missingness were removed from the initial candidate pool. For the final 13 selected predictors, the exported modeling dataset was highly complete (0% missingness).

### 2.3 Feature selection

Feature selection was performed strictly within the training set using cross-validated recursive feature elimination (RFECV). A random forest classifier (n_estimators = 100, random_state = 696) was used as the base estimator, with step size set to 1 and 5-fold cross-validation used to evaluate cross-validated AUC. Thirteen predictors were retained for final model development: proBNP, HCT, TBil, DBil, BUN, *β*2-MG, CRP, UA, Glb, GGT, MCH, LDL-C, and FIB.

### 2.4 Model development and tuning

Six classifiers were developed and compared: logistic regression (LR), random forest (RF), extreme gradient boosting (XGB), support vector machine (SVM), k-nearest neighbors (KNN), and multilayer perceptron (MLP). Hyperparameters were optimized using grid search with cross-validation within the training set, with AUC as the primary optimization metric. The final optimized hyperparameters are listed in Supplementary Table S3.

### 2.5 Model evaluation and comparison

Discrimination was assessed using receiver operating characteristic (ROC) curves and AUCs. Sensitivity and specificity at the default classification threshold were calculated in the independent test set, and 95% confidence intervals were estimated by bootstrap resampling. Calibration was evaluated using calibration curves, Brier score, calibration intercept, and calibration slope. Pairwise test-set AUC differences were assessed using bootstrap resampling. Because HFrEF represented 25.5% of the cohort, precision-recall analysis was additionally performed. Clinical utility was evaluated using decision-curve analysis, and threshold-moving analysis was conducted to support the intended action of prioritized echocardiography or HF specialist referral rather than immediate treatment initiation.

### 2.6 Incremental value, interpretation, and deployment

To evaluate the incremental value of multisystem laboratory integration, the final RF model was compared with a proBNP-only benchmark and a clinical baseline model including proBNP, age, sex, and BUN. Continuous net reclassification improvement (NRI) and integrated discrimination improvement (IDI) were calculated. SHAP (SHapley Additive exPlanations) was used to quantify global feature importance and visualize nonlinear feature-effect relationships for the RF model. Standardized LR odds ratios were additionally extracted to provide a transparent regression-based interpretation. A web-based application was developed to support individualized probability estimation, and a prominent disclaimer was included indicating that the tool is for academic research only and must not replace clinical judgment pending external validation.

### 2.7 Statistical analysis

Analyses were conducted in Python 3.10 using NumPy, Pandas, SciPy, scikit-learn 1.2, XGBoost 1.7, SHAP, statsmodels, Matplotlib, and Seaborn. Continuous variables are presented as mean ± standard deviation in the baseline table. Differences between groups were assessed using Student's t-test for normally distributed continuous variables, the Mann–Whitney U test for skewed continuous variables, and the chi-square test for categorical variables, as appropriate. All statistical tests were two-sided, and *P* < 0.05 was considered statistically significant.

## 3 Results

### 3.1 Patient characteristics

A total of 1,480 hospitalized patients with chronic HF were included, of whom 377 (25.5%) had HFrEF and 1,103 (74.5%) had HFmrEF/HFpEF. The overall mean age was 74.92 ± 8.16 years. Baseline characteristics are summarized in Table 1. Compared with the HFmrEF/HFpEF group, patients with HFrEF had higher mean levels of proBNP (12,107.05 ± 10,369.10 vs. 5,437.78 ± 7,817.20 pg/mL), BUN (8.18 ± 4.30 vs. 7.45 ± 5.15 mmol/L), TBil (16.86 ± 16.30 vs. 12.48 ± 10.69 *μ*mol/L), DBil (7.84 ± 11.14 vs. 5.49 ± 6.89 *μ*mol/L), GGT (69.25 ± 73.66 vs. 47.95 ± 58.47 U/L), HCT (37.59 ± 5.40% vs. 35.61 ± 7.23%), and UA (474.61 ± 148.75 vs. 415.24 ± 142.71 *μ*mol/L), while *β*2-MG, CRP, Glb, TP, PLT, and FIB were numerically lower. These differences support a multisystem laboratory signature associated with reduced ejection fraction.

**Table 1:** Baseline characteristics of patients with HFmrEF/HFpEF and HFrEF.

Clinical factors	Overall (*N* = 1,480)	HFmrEF/HFpEF (*N* = 1,103)	HFrEF (*N* = 377)
gender_male	811	564	247
gender_female	669	539	130
Age, years (Mean ± SD)	74.92 ± 8.16	75.17 ± 8.01	74.18 ± 8.55
proBNP, pg/mL (Mean ± SD)	7,136.64 ± 9,017.52	5,437.78 ± 7,817.20	12,107.05 ± 10,369.10
CRE, μmol/L (Mean ± SD)	118.67 ± 111.94	119.46 ± 122.50	116.36 ± 72.75
BUN, mmol/L (Mean ± SD)	7.64 ± 4.96	7.45 ± 5.15	8.18 ± 4.30
UA, μmol/L (Mean ± SD)	430.37 ± 146.53	415.24 ± 142.71	474.61 ± 148.75
Cys_C, mg/L (Mean ± SD)	1.59 ± 0.75	1.59 ± 0.78	1.59 ± 0.66
*β*_2_-MG, mg/L (Mean ± SD)	3.95 ± 3.37	4.06 ± 3.57	3.62 ± 2.70
Na, mmol/L (Mean ± SD)	139.33 ± 4.72	139.29 ± 4.88	139.43 ± 4.23
K, mmol/L (Mean ± SD)	4.01 ± 0.64	3.99 ± 0.65	4.09 ± 0.60
Cl, mmol/L (Mean ± SD)	102.96 ± 6.07	102.85 ± 6.25	103.28 ± 5.52
Ca, mmol/L (Mean ± SD)	2.20 ± 0.14	2.20 ± 0.15	2.21 ± 0.13
P, mmol/L (Mean ± SD)	1.12 ± 0.33	1.10 ± 0.33	1.16 ± 0.33
Hb, g/L (Mean ± SD)	118.18 ± 23.84	116.37 ± 24.95	123.47 ± 19.35
RBC, ×10^12^/L (Mean ± SD)	4.24 ± 0.87	4.20 ± 0.91	4.35 ± 0.75
HCT, % (Mean ± SD)	36.11 ± 6.86	35.61 ± 7.23	37.59 ± 5.40
MCH, pg (Mean ± SD)	28.23 ± 4.19	28.05 ± 4.21	28.73 ± 4.07
PLT, ×10⁹/L (Mean ± SD)	240.80 ± 81.96	244.34 ± 82.84	230.47 ± 78.51
CRP, mg/L (Mean ± SD)	23.80 ± 37.86	25.82 ± 41.10	17.88 ± 25.34
WBC, ×10^9^/L (Mean ± SD)	8.74 ± 4.11	8.81 ± 4.28	8.55 ± 3.57
Neutrophil % (Mean ± SD)	73.94 ± 12.52	73.71 ± 12.70	74.62 ± 11.97
Monocyte % (Mean ± SD)	7.72 ± 3.61	7.81 ± 3.74	7.45 ± 3.18
TBil, μmol/L (Mean ± SD)	13.59 ± 12.50	12.48 ± 10.69	16.86 ± 16.30
DBil, μmol/L (Mean ± SD)	6.09 ± 8.25	5.49 ± 6.89	7.84 ± 11.14
ALT, U/L (Mean ± SD)	37.90 ± 102.15	31.46 ± 81.07	56.76 ± 145.96
AST, U/L (Mean ± SD)	51.67 ± 177.87	42.66 ± 130.46	78.05 ± 271.35
GGT, U/L (Mean ± SD)	53.38 ± 63.35	47.95 ± 58.47	69.25 ± 73.66
Glb, g/L (Mean ± SD)	26.65 ± 5.57	26.98 ± 5.66	25.70 ± 5.19
TP, g/L (Mean ± SD)	64.30 ± 6.80	64.57 ± 6.89	63.53 ± 6.50
CK, U/L (Mean ± SD)	163.55 ± 244.90	159.89 ± 248.36	174.27 ± 234.47
CK-MB, U/L (Mean ± SD)	21.84 ± 21.09	21.59 ± 21.84	22.57 ± 18.74
LDH, U/L (Mean ± SD)	284.54 ± 212.55	274.53 ± 174.80	313.80 ± 294.97
PT, seconds (Mean ± SD)	12.52 ± 5.59	12.37 ± 6.02	12.96 ± 4.08
INR (Mean ± SD)	1.06 ± 0.30	1.04 ± 0.28	1.10 ± 0.35
FIB, g/L (Mean ± SD)	3.53 ± 2.18	3.62 ± 2.40	3.27 ± 1.32
APTT, seconds (Mean ± SD)	29.89 ± 11.45	29.66 ± 11.72	30.55 ± 10.58
TC, mmol/L (Mean ± SD)	4.22 ± 1.20	4.21 ± 1.24	4.25 ± 1.07
TG, mmol/L (Mean ± SD)	1.35 ± 0.92	1.38 ± 0.92	1.28 ± 0.93
HDL-C, mmol/L (Mean ± SD)	1.12 ± 0.35	1.12 ± 0.35	1.11 ± 0.33
LDL-C, mmol/L (Mean ± SD)	2.52 ± 0.96	2.49 ± 0.99	2.61 ± 0.87

Values are presented as mean ± standard deviation unless otherwise indicated. HFrEF, heart failure with reduced ejection fraction; HFmrEF, heart failure with mildly reduced ejection fraction; HFpEF, heart failure with preserved ejection fraction; proBNP, pro-B-type natriuretic peptide; CRE, creatinine; BUN, blood urea nitrogen; UA, uric acid; Cys-C, cystatin C; β2-MG, beta2-microglobulin; Hb, hemoglobin; RBC, red blood cell count; HCT, hematocrit; MCH, mean corpuscular hemoglobin; PLT, platelet count; CRP, C-reactive protein; WBC, white blood cell count; TBil, total bilirubin; DBil, direct bilirubin; ALT, alanine aminotransferase; AST, aspartate aminotransferase; GGT, gamma-glutamyl transferase; Glb, globulin; TP, total protein; CK-MB, creatine kinase-MB; LDH, lactate dehydrogenase; PT, prothrombin time; INR, international normalized ratio; FIB, fibrinogen; APTT, activated partial thromboplastin time; TC, total cholesterol; TG, triglycerides; HDL-C, high-density lipoprotein cholesterol; LDL-C, low-density lipoprotein cholesterol. Group comparisons were performed using Student's t-test, Mann–Whitney U test, or chi-square test as appropriate.

### 3.2 Selected features

**Figure 2:**
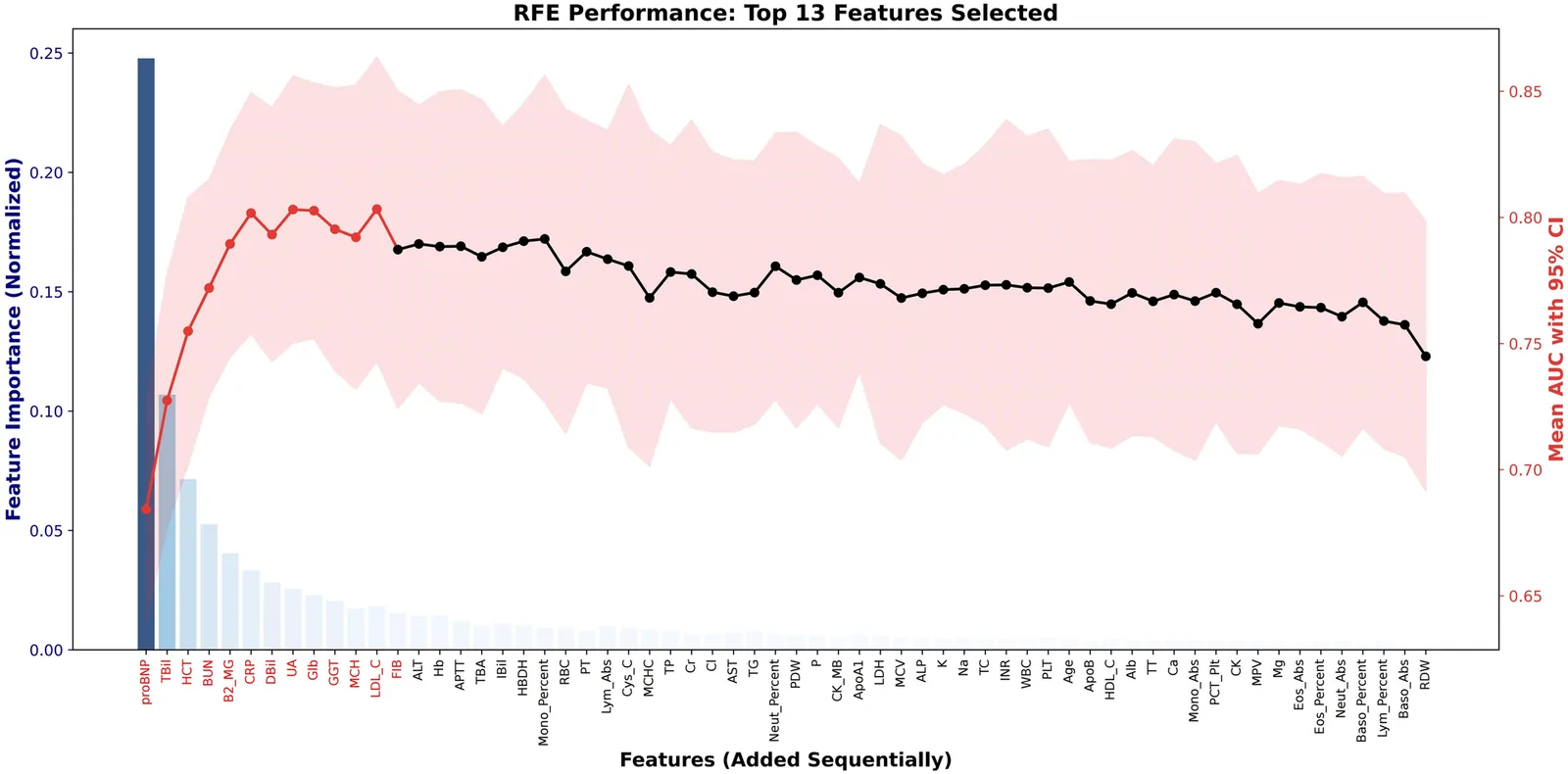
Cross-validated recursive feature elimination for routine laboratory predictors. Mean cross-validated AUC was used to evaluate candidate feature subsets. The selected 13-indicator panel balances parsimony and predictive performance. The shaded region represents variability across cross-validation folds. AUC, area under the receiver operating characteristic curve.

RFECV identified a parsimonious 13-indicator panel for subsequent modeling (Figure 2). The final predictor set comprised proBNP, HCT, TBil, DBil, BUN, *β*2-MG, CRP, UA, Glb, GGT, MCH, LDL-C, and FIB. This panel integrates markers of myocardial stress, hemoconcentration or hematologic status, hepatobiliary congestion, renal function, inflammation, lipid metabolism, and coagulation.

### 3.3 Comparative model performance and threshold optimization

**Figure 3:**
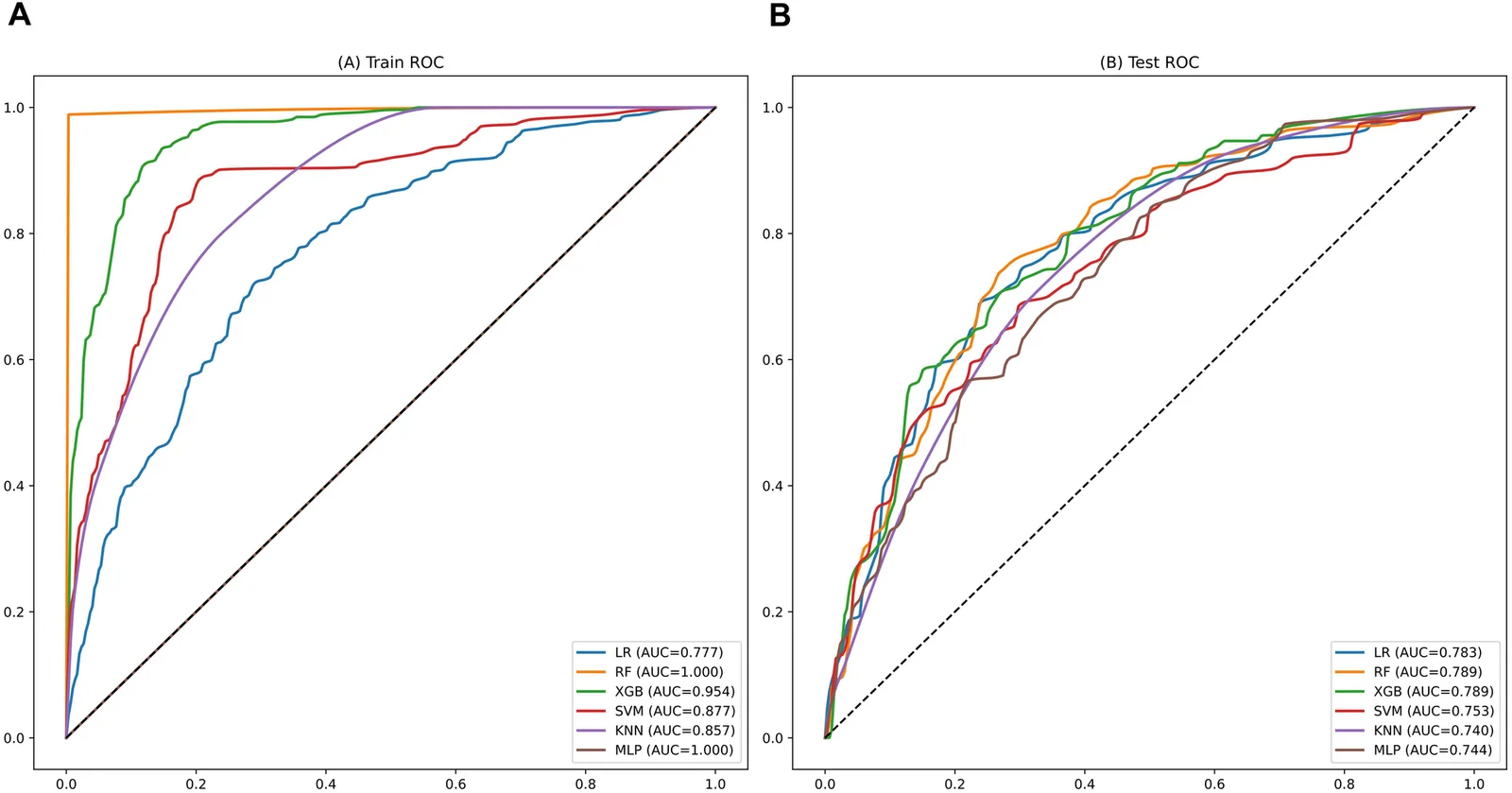
Receiver operating characteristic curves of the six machine-learning models in the training and test sets. **(A)** Training set. **(B)** Test set. Each curve shows sensitivity vs. 1-specificity. The dashed diagonal line represents a random classifier (AUC = 0.5). AUC values are shown in the legends. ROC, receiver operating characteristic; AUC, area under the ROC curve.

Model performance is summarized in Table 2 and Figure 3. In the independent test set, RF and XGB achieved the highest AUCs (both 0.789), followed closely by LR (0.784), SVM (0.753), MLP (0.743), and KNN (0.739). RF had the lowest Brier score (0.152), but the absolute calibration difference from LR (Brier score 0.155) was small. At the default threshold, RF and XGB favored specificity (0.931 and 0.979, respectively) at the expense of sensitivity (0.320 and 0.134), whereas MLP and KNN yielded higher sensitivity (0.494 and 0.425) but lower specificity.

**Table 2:** Performance of the machine-learning models in the test set.

Model	AUC (95% CI)	SENS (95% CI)	SPEC (95% CI)	Brier (95% CI)	Calib_Intercept	Calib_Slope
LR	0.784 (0.733–0.831)	0.256 (0.175–0.336)	0.934 (0.907–0.958)	0.155 (0.137–0.174)	0.017	1.069
RF	0.789 (0.744–0.836)	0.320 (0.236–0.408)	0.931 (0.904–0.956)	0.152 (0.134–0.171)	−0.046	1.076
XGB	0.789 (0.743–0.833)	0.134 (0.075–0.198)	0.979 (0.962–0.994)	0.157 (0.141–0.174)	0.379	1.600
SVM	0.753 (0.697–0.803)	0.219 (0.143–0.296)	0.960 (0.939–0.979)	0.161 (0.141–0.180)	0.175	1.191
KNN	0.739 (0.689–0.785)	0.425 (0.330–0.515)	0.852 (0.813–0.890)	0.170 (0.151–0.191)	−0.637	0.173
MLP	0.743 (0.693–0.792)	0.494 (0.402–0.585)	0.806 (0.762–0.846)	0.234 (0.201–0.270)	−0.617	0.131

AUC, sensitivity, specificity, and Brier score are reported with bootstrap 95% confidence intervals. LR, logistic regression; RF, random forest; XGB, extreme gradient boosting; SVM, support vector machine; KNN, k-nearest neighbors; MLP, multilayer perceptron; AUC, area under the receiver operating characteristic curve; SENS, sensitivity; SPEC, specificity; Brier, Brier score; Calib, calibration.

Because missing HFrEF would be clinically undesirable when the intended action is prioritized echocardiography, threshold optimization was performed for the RF model (Supplementary Table S1). A threshold of 0.15 achieved sensitivity of 0.912 and negative predictive value of 0.935, indicating that a low threshold is more appropriate for rule-out triage than the default threshold. At this threshold, the model would prioritize most true HFrEF cases for imaging or specialist review, while accepting additional false positives as the trade-off for avoiding missed HFrEF.

The incremental value of the multimarker RF model was assessed against benchmark models (Supplementary Table S2). Compared with the proBNP-only model (AUC: 0.767), the 13-variable RF model (AUC: 0.789) improved reclassification and discrimination (continuous NRI: 0.5346; IDI: 0.0873). Compared with the clinical baseline model (proBNP + age + sex + BUN; AUC: 0.743), the RF model also improved performance (continuous NRI: 0.4332; IDI: 0.0750), supporting additive value from routine multisystem indicators beyond natriuretic peptides alone.

### 3.4 Calibration, pairwise comparison, and clinical utility

**Figure 4:**
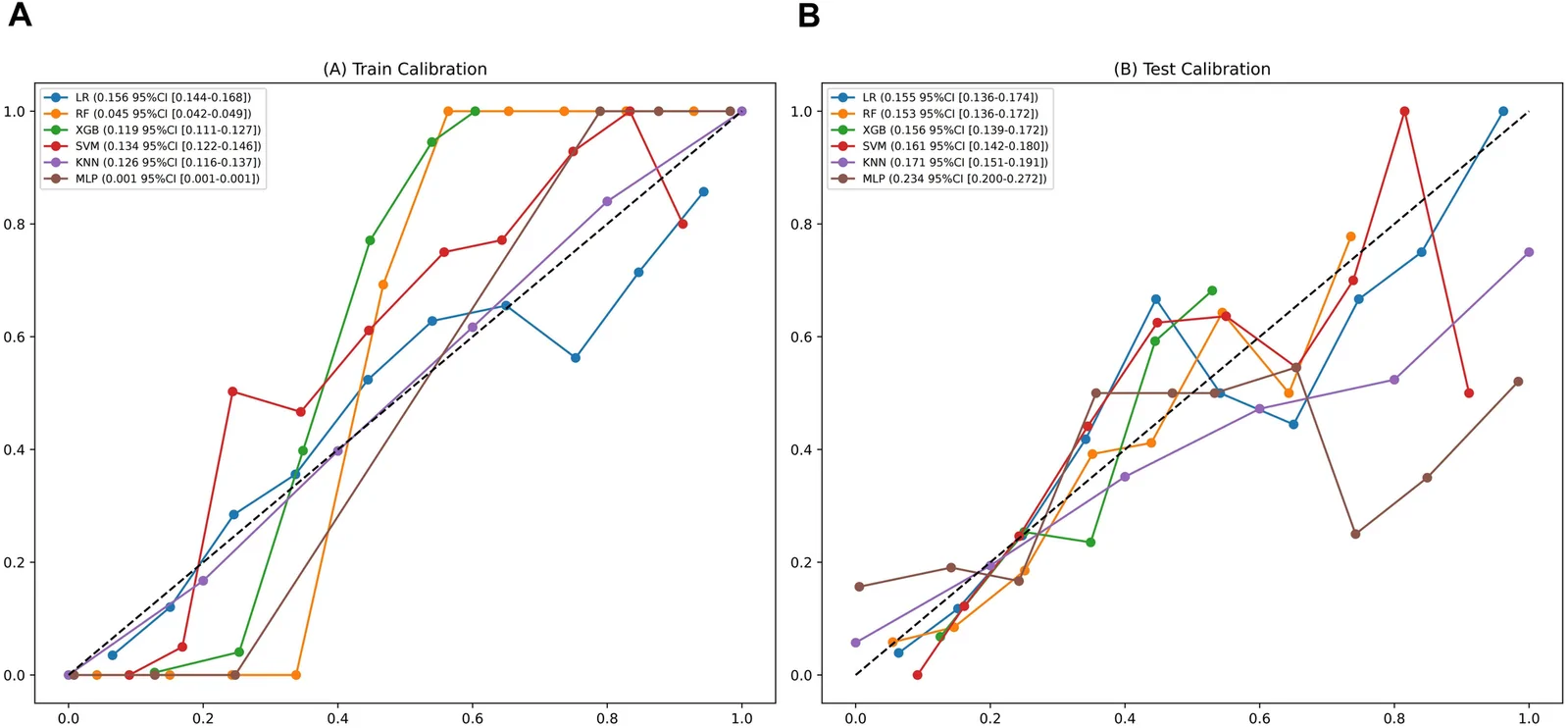
Calibration curves of the six machine-learning models in the training and test sets. **(A)** Training set. **(B)** Test set. The diagonal line indicates perfect agreement between predicted probabilities and observed event rates. Calibration was summarized using Brier score, calibration intercept, and calibration slope.

**Figure 5:**
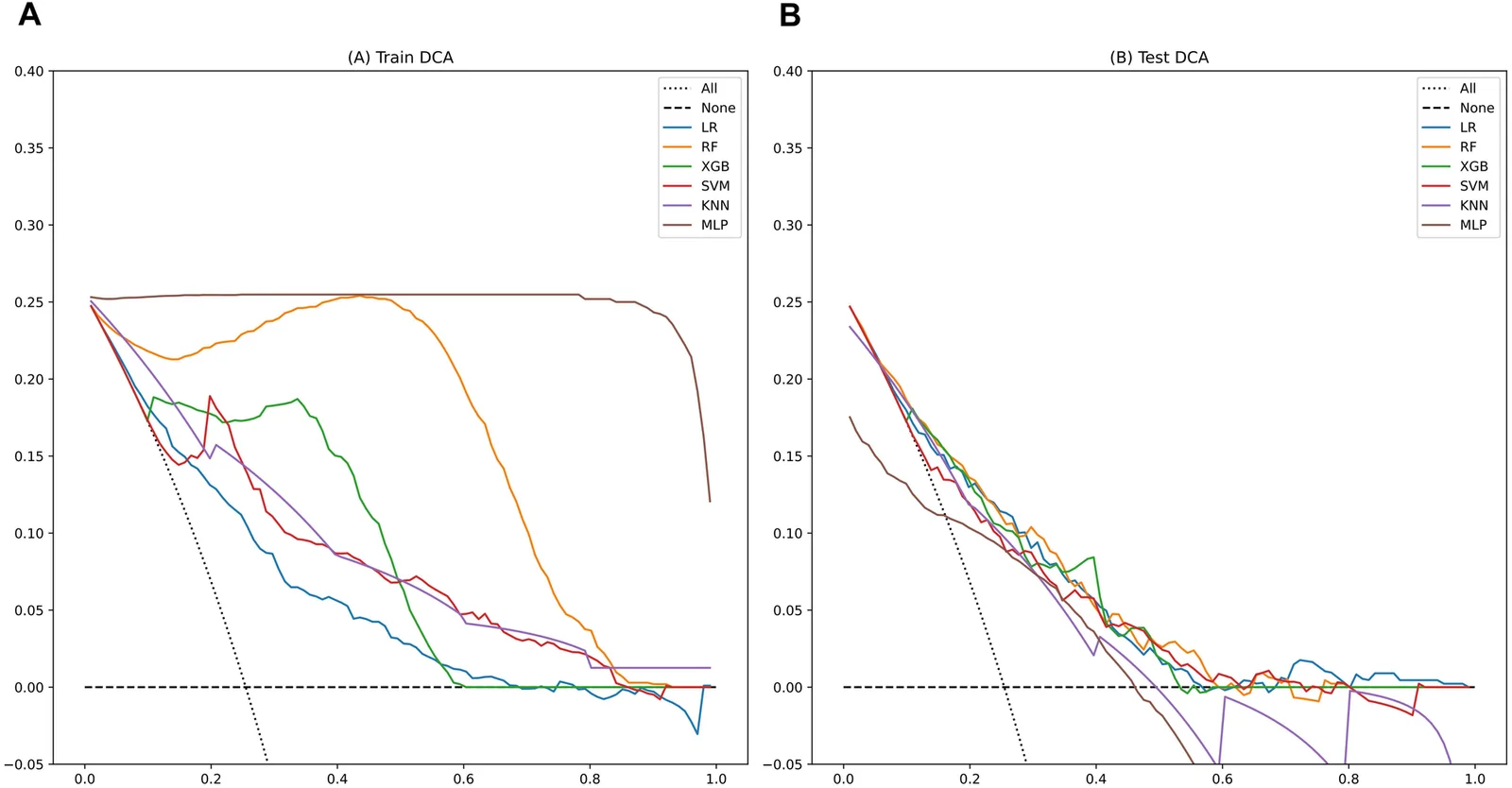
Decision-curve analysis of the six machine-learning models in the training and test sets. **(A)** Training set. **(B)** Test set. Net benefit is plotted against threshold probability. Treat-all and treat-none strategies are shown as reference lines. For the present triage scenario, positive predictions are intended to trigger prioritized echocardiography or specialist referral, not treatment initiation before imaging confirmation.

**Figure 6:**
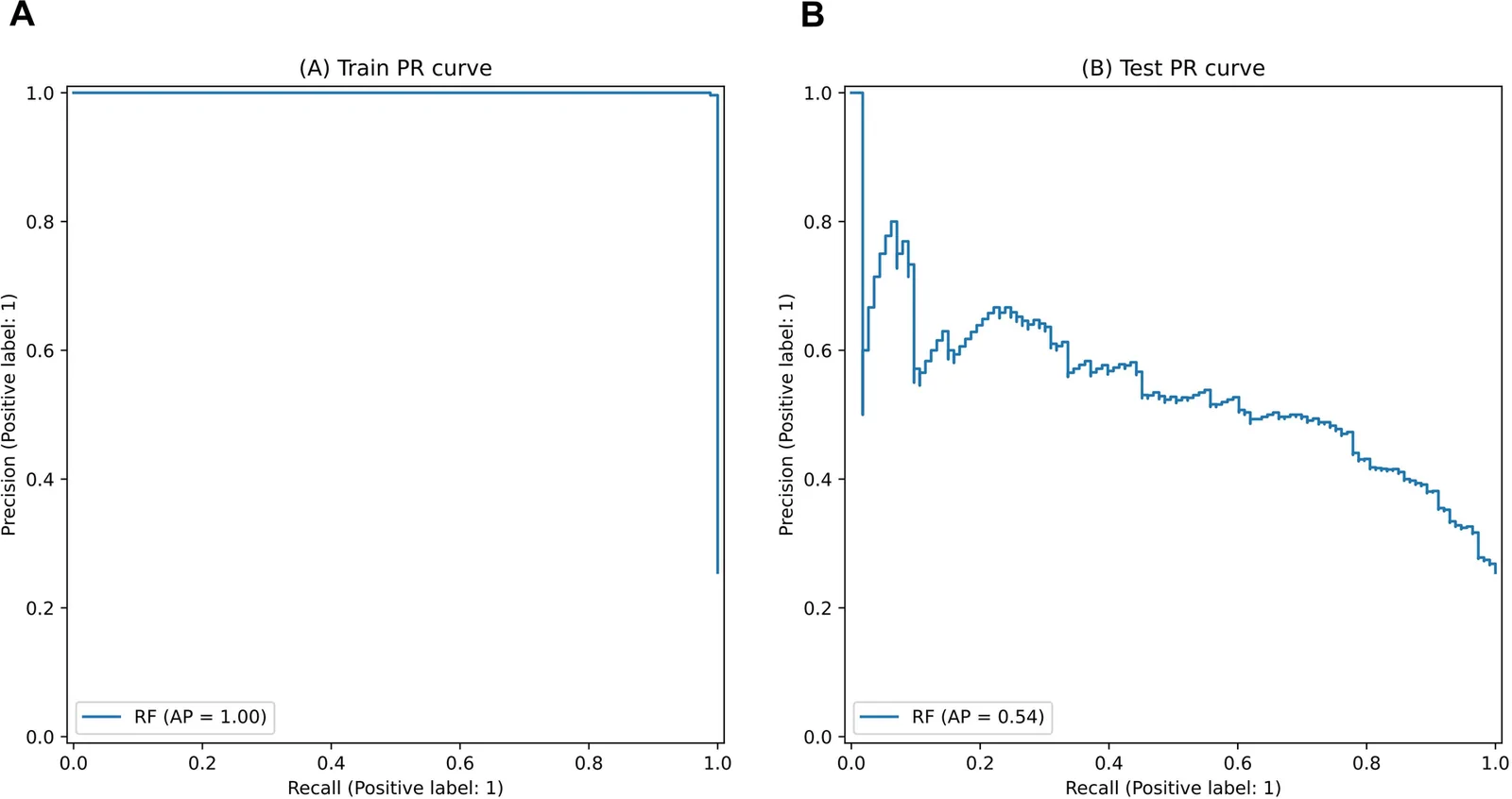
Precision-recall curves of the final random forest model in the training and test sets. **(A)** Training set. **(B)** Test set. Precision is plotted against recall across thresholds. The curves illustrate performance under class imbalance, where HFrEF accounted for 25.5% of the cohort.

Pairwise bootstrap comparisons of test-set AUCs showed no statistically significant differences among models (all *P* > 0.05; Table 3). Therefore, RF should not be interpreted as clearly superior to simpler alternatives. Calibration evaluation showed that LR had near-ideal calibration-in-the-large and spread (intercept 0.017; slope 1.069), whereas RF had the numerically lowest Brier score (Figure 4; Table 2). Decision-curve analysis indicated that RF and XGB yielded greater net benefit than treat-all or treat-none strategies over clinically relevant low-to-moderate threshold probabilities, particularly when the action is prioritized echocardiography or specialist referral rather than immediate HFrEF-specific treatment (Figure 5). Precision-recall analysis further characterized the trade-off between positive predictive value and recall under class imbalance (Figure 6).

**Table 3:** Pairwise model comparison using bootstrap *P* values.

Comparison	AUC_1	AUC_2	*P*_Value
LR vs. RF	0.783	0.789	0.7225
LR vs. XGB	0.783	0.789	0.7405
LR vs. SVM	0.783	0.753	0.504
LR vs. KNN	0.783	0.74	0.503
LR vs. MLP	0.783	0.744	0.505
RF vs. XGB	0.789	0.789	0.9675
RF vs. SVM	0.789	0.753	0.492
RF vs. KNN	0.789	0.74	0.4985
RF vs. MLP	0.789	0.744	0.4845
XGB vs. SVM	0.789	0.753	0.4925
XGB vs. KNN	0.789	0.74	0.4895
XGB vs. MLP	0.789	0.744	0.4885
SVM vs. KNN	0.753	0.74	0.647
SVM vs. MLP	0.753	0.744	0.7055
KNN vs. MLP	0.74	0.744	0.8875

All *P* values were derived from bootstrap-based pairwise comparisons of test-set AUCs. A two-sided *P* < 0.05 was considered statistically significant.

### 3.5 Subgroup and phenotype-sensitivity analyses

To examine robustness against potential demographic and structural confounding, the RF model was evaluated across prespecified test-set subgroups. The model maintained discrimination across age strata (AUC: 0.780 for age ≥65 years and 0.863 for age <65 years) and BUN strata (AUC: 0.769 for BUN ≥ 6.4 mmol/L and 0.793 for BUN < 6.4 mmol/L), as shown in Supplementary Figure S1. In a phenotype-sensitivity analysis that excluded HFmrEF from the control group, the HFrEF-vs.-HFpEF classification task yielded an AUC of 0.829 (Supplementary Table S4), suggesting that the model retained discrimination when the non-HFrEF comparator was restricted to HFpEF.

### 3.6 Model interpretation and transparent benchmarks

**Figure 7:**
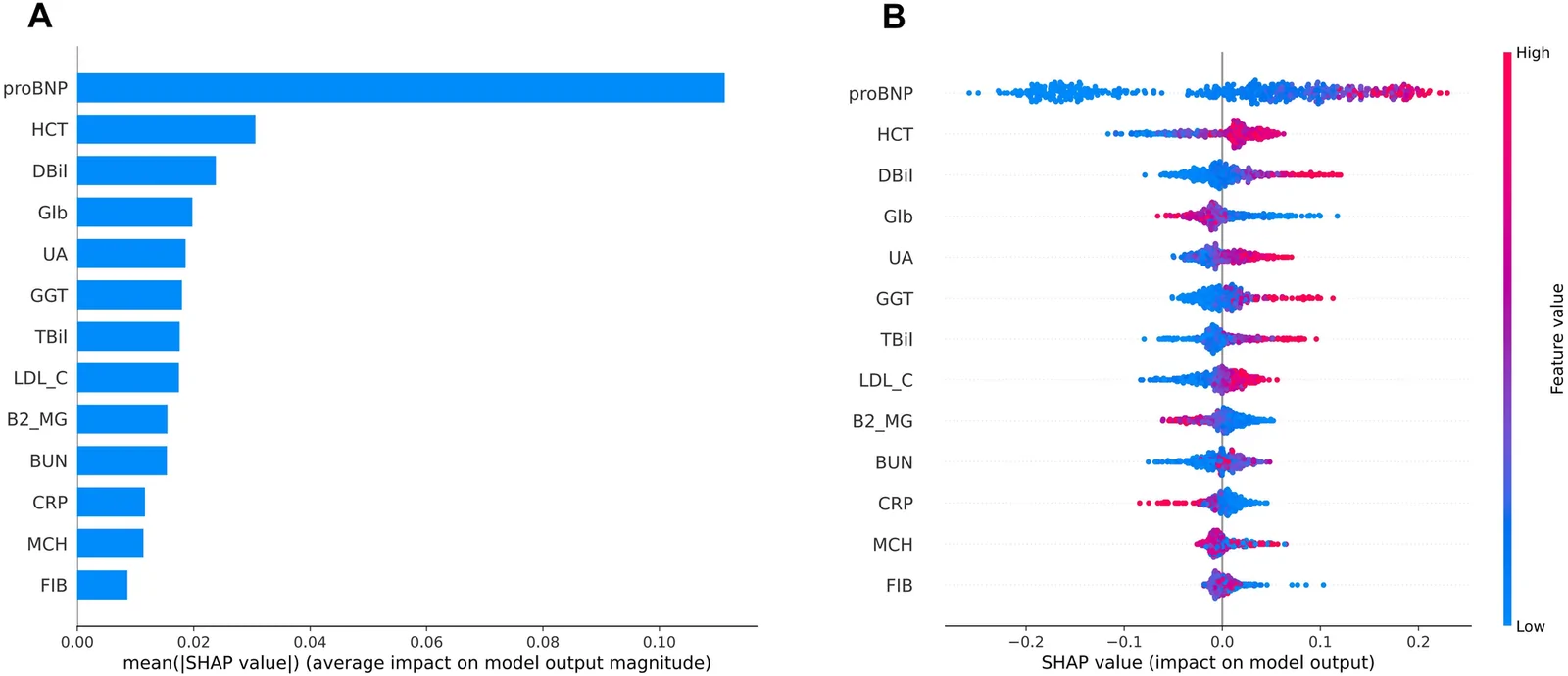
SHAP summary plots for the random forest model. **(A)** Feature importance ranking by mean absolute SHAP values. **(B)** SHAP beeswarm plot. Each dot represents one patient; color indicates feature value; horizontal position indicates the direction and magnitude of contribution to HFrEF probability. SHAP, SHapley Additive exPlanations.

**Figure 8:**
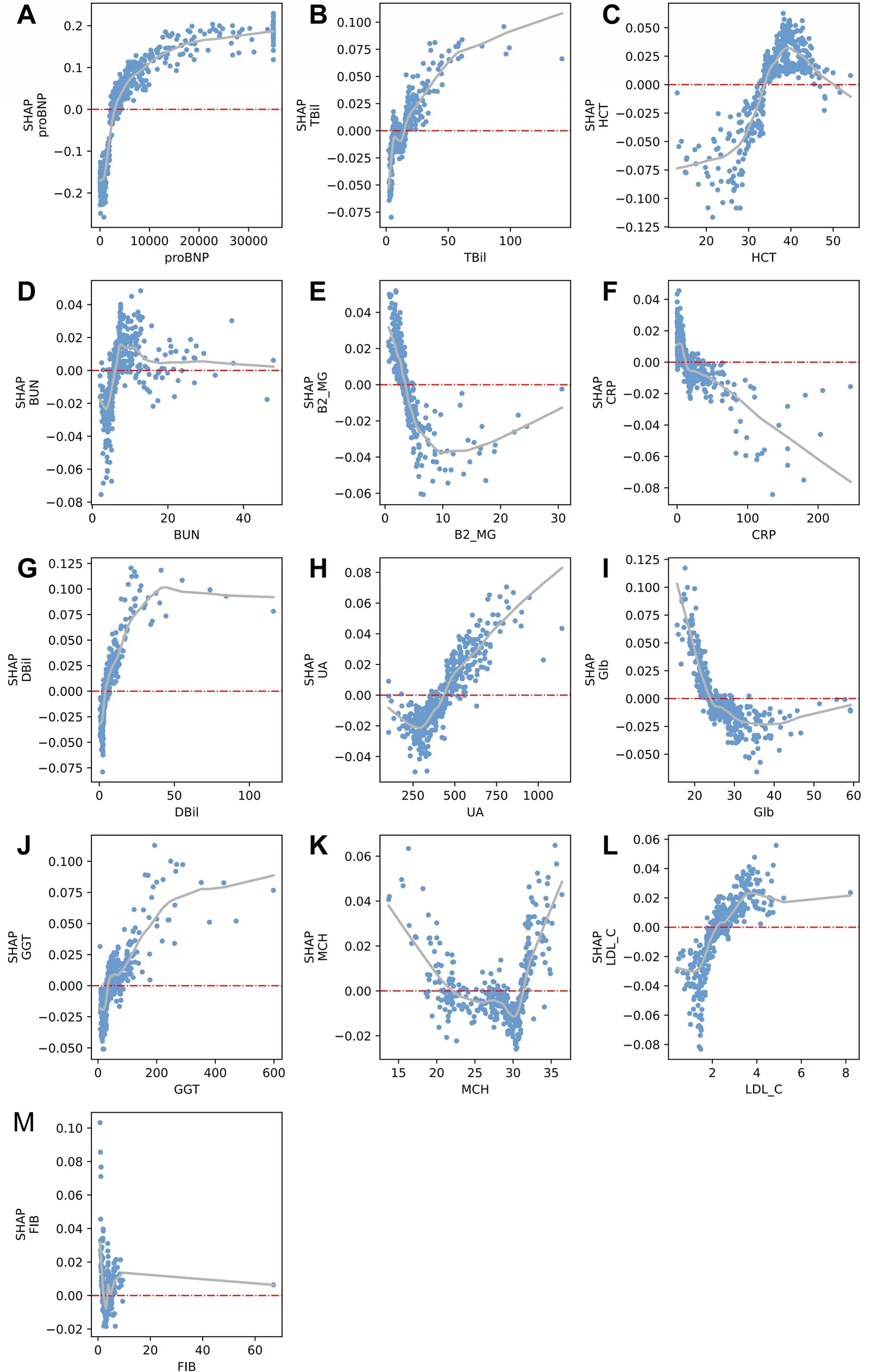
SHAP dependence plots (**A-M**) for the 13 selected predictors Each panel shows the SHAP value as a function of the corresponding predictor value. LOWESS-smoothed curves illustrate nonlinear predictor effects on HFrEF probability. The 13 selected predictors were proBNP, HCT, TBil, DBil, BUN, *β*2-MG, CRP, UA, Glb, GGT, MCH, LDL-C, and FIB.

SHAP analysis of the RF model identified proBNP as the dominant contributor to HFrEF prediction, with a mean absolute SHAP value of 0.111 (Supplementary Table S5; Figures 7, 8). The next most influential variables were HCT (0.031), DBil (0.024), Glb (0.020), UA (0.019), GGT (0.018), TBil (0.018), and LDL-C (0.017), followed by *β*2-MG, BUN, CRP, MCH, and FIB. Feature-effect plots suggested a clear positive association between proBNP and predicted HFrEF probability, whereas several hepatic, renal, inflammatory, lipid, and coagulation markers showed nonlinear associations.

To complement the SHAP analysis, standardized LR odds ratios were extracted (Supplementary Figure S2). In the linear benchmark model, proBNP (OR: 2.40), HCT (OR: 1.34), and DBil (OR: 1.22) were among the strongest positive predictors, supporting the biological plausibility of the selected feature set while preserving the interpretability of LR as a clinically transparent comparator.

### 3.7 Web-based application

**Figure 9:**
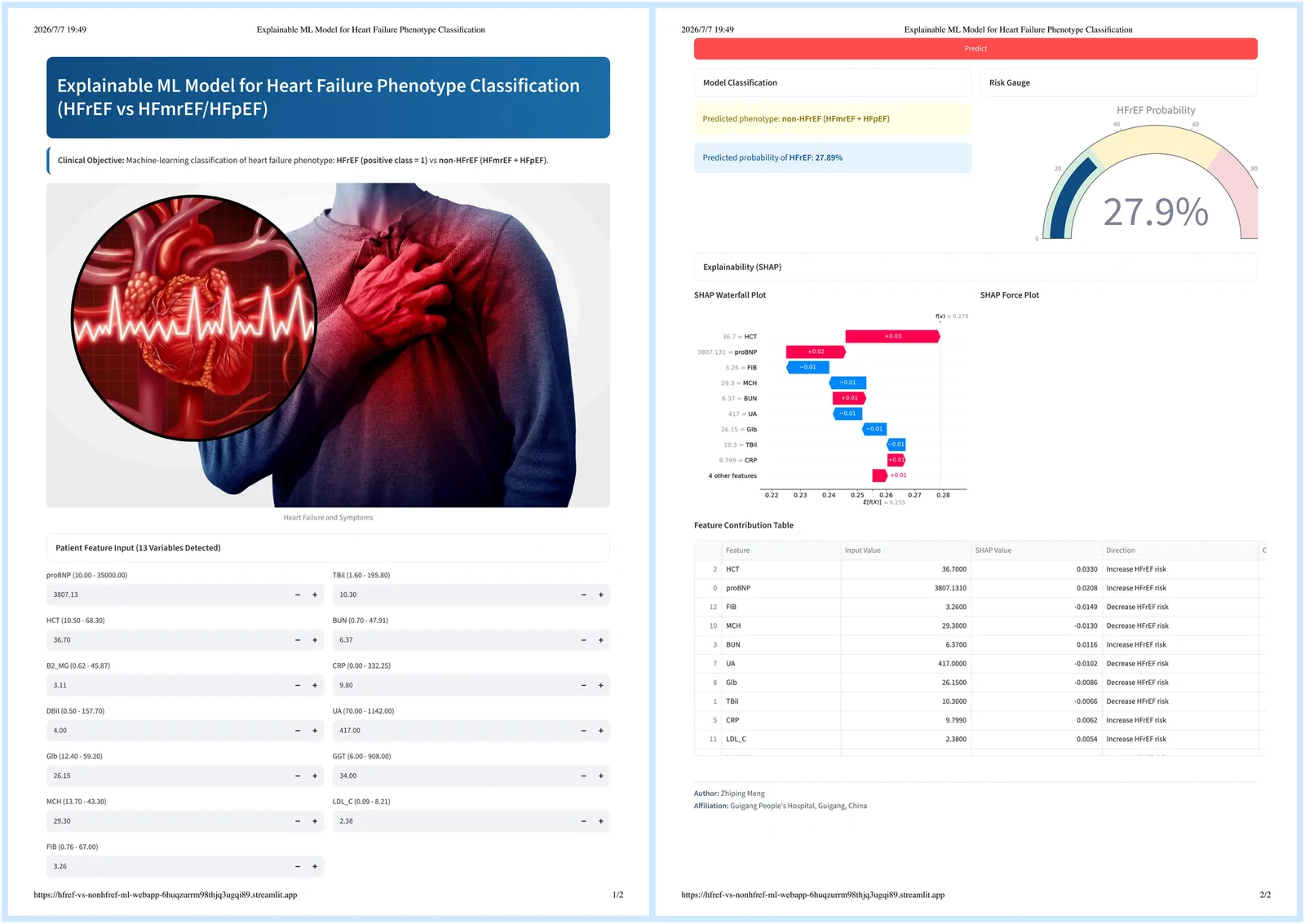
Representative interface of the web-based calculator for individualized HFrEF prediction. The interface allows entry of the 13 selected laboratory indicators and returns an individualized HFrEF probability with feature-level explanations. The calculator is intended for academic research and requires external validation before clinical use.

A web-based calculator (accessible at: https://hfref-vs-nonhfref-ml-webapp-6huqzurrm98thjq3ugqi89.streamlit.app) was developed to generate individualized HFrEF probability estimates from the 13 selected laboratory variables (Figure 9). The interface supports real-time data entry and returns a predicted probability with feature-level explanations. The application includes an explicit research-use disclaimer and should not be used as a stand-alone clinical decision tool before external validation.

## 4 Discussion

### 4.1 Principal findings and clinical interpretation

This study developed and internally evaluated a routine laboratory-based machine-learning approach for distinguishing HFrEF from HFmrEF/HFpEF in hospitalized patients with chronic HF. Three findings are central. First, a 13-indicator laboratory panel that captures myocardial stress, hepatobiliary congestion, renal function, hematologic status, inflammation, lipid metabolism, and coagulation achieved moderate discrimination in a leakage-free workflow. Second, RF and XGB had the highest numerical AUCs, but LR performed similarly and had excellent calibration; thus, complex machine-learning models cannot be claimed to be statistically superior without external validation. Third, default-threshold classification was not suitable for screening because sensitivity was limited. A lower threshold of 0.15 transformed the RF model into a more appropriate rule-out triage tool for prioritized echocardiography, with sensitivity > 91% and NPV > 93%.

The intended clinical action is crucial for interpreting the model. This tool is not designed to initiate HFrEF-specific treatment before echocardiography. Instead, a positive result at a low threshold should trigger prioritized echocardiography, repeat natriuretic peptide assessment, or HF specialist evaluation. Because these actions carry relatively low direct risk compared with delayed recognition of HFrEF, a low threshold probability is clinically reasonable. Decision-curve analysis is therefore best interpreted in the context of triage and resource allocation, not as justification for replacing imaging-based phenotyping.

### 4.2 Biological plausibility of the selected variables

Natriuretic peptides remain among the best validated biomarkers for HF diagnosis and risk stratification, and recent evidence supports their prognostic value in HFpEF as well (6, 15). Nevertheless, their interpretation can be modified by obesity, kidney function, and functional capacity (6–8). In the present model, proBNP had the largest SHAP contribution, but the incremental-value analysis suggests that multisystem variables add clinically relevant information to a proBNP-only strategy.

The contribution of hepatic markers is consistent with cardiohepatic interaction in HF. Liver biomarkers have been associated with prognosis in HFpEF, while hypoalbuminemia and nutrition-associated markers reflect chronic inflammatory, nutritional, and congestion-related burden (16–18). Bilirubin abnormalities, particularly direct bilirubin, have also been associated with adverse HF outcomes (19). Accordingly, DBil, TBil, GGT, and Glb should be interpreted as composite signals of congestion, cholestasis, oxidative stress, and inflammatory or nutritional status rather than as causal determinants of EF phenotype.

Renal function and systemic congestion were represented by BUN and *β*2-MG. Systematic evidence links BUN and the BUN-to-creatinine ratio to adverse outcomes in HF, likely reflecting renal perfusion, neurohormonal activation, and volume status (20, 21). *β*2-MG is also related to renal dysfunction, inflammatory activation, and vascular remodeling, and proteomic evidence has implicated B2M in HFpEF complicated by pulmonary hypertension (22). These findings support the inclusion of renal and inflammatory markers while also underscoring the need to interpret them in clinical context.

Hematologic and inflammatory markers further contributed to model behavior. Anemia has been consistently associated with mortality and rehospitalization in HF, including acute HF and HFpEF cohorts (23–25). HCT may therefore capture anemia, hemodilution, sex-related differences, or volume status. CRP and monocyte-related immune activity are likewise linked to HF heterogeneity and outcomes, and inflammatory profiles differ between HFpEF and HFrEF (26–29). These markers add biological plausibility, but they are also susceptible to confounding by infection, comorbidity, and acute decompensation.

### 4.3 Methodological contribution and comparison with benchmarks

From a methodological perspective, the study demonstrates the importance of leakage-free preprocessing, transparent benchmark models, calibration assessment, and threshold-specific reporting in clinical prediction modeling. Machine learning may capture nonlinear patterns that conventional regression does not fully represent, but explainability and calibration are equally important for clinical translation. Recent HF prediction studies have shown that explainable machine learning and online calculators can be implemented for readmission and mortality prediction (30, 31). In the present study, SHAP and standardized LR odds ratios were used together to balance nonlinear interpretation with clinically familiar regression-based transparency.

The comparison with proBNP-only and clinical baseline models is particularly important. Although proBNP dominated feature importance, the 13-variable RF model improved NRI and IDI over both benchmarks. This supports the concept that multisystem laboratory integration captures phenotypic information beyond natriuretic peptide concentration alone. However, because pairwise AUC differences among algorithms were not significant and LR showed excellent calibration, future deployment should consider simplicity, transportability, and recalibration rather than focusing solely on the highest numerical AUC. Decision-curve analysis can complement discrimination and calibration by quantifying net clinical benefit across thresholds (30–32).

### 4.4 Phenotype heterogeneity and limitations

Combining HFmrEF and HFpEF into a single non-HFrEF group supports a binary triage objective, but it inevitably introduces phenotypic heterogeneity. HFmrEF can overlap with HFrEF in pathophysiology, treatment responsiveness, and prognosis. The HFrEF-vs.-HFpEF sensitivity analysis yielded an AUC of 0.829, suggesting that discrimination persisted when HFmrEF was excluded. However, future studies should explicitly model HFmrEF, HFpEF, HFrEF, and HFimpEF using prior LVEF trajectories, etiology, rhythm status, medication exposure, and longitudinal outcome data.

Several limitations should be acknowledged. First, this was a retrospective single-center study, and the sample composition, testing workflow, and local echocardiography practices may limit generalizability. Second, the model was built primarily on routine laboratory variables and did not incorporate echocardiographic structure, treatment information, BMI, atrial fibrillation, etiology, or longitudinal follow-up. Third, although laboratory sampling was anchored to the first 24 h of admission, residual confounding by disease severity, volume status, comorbidity, and early treatment cannot be excluded. Fourth, the database did not consistently retain prior LVEF values, measurement-method details, or complete HFimpEF definitions. Fifth, despite internal validation, the model requires multicenter external validation and prospective workflow evaluation to mitigate risk of bias and assess applicability under PROBAST + AI principles (14).

## 5 Conclusion

A machine-learning model constructed from 13 routine laboratory indicators distinguished HFrEF from HFmrEF/HFpEF with moderate discrimination in a single-center retrospective cohort. RF and XGB had the highest numerical AUCs (both 0.789), and RF had the lowest Brier score, but no algorithm demonstrated statistically superior AUC. After threshold optimization to 0.15, the RF model achieved sensitivity of 0.912 and NPV of 0.935, supporting its potential as an adjunctive rule-out triage tool for prioritized echocardiography or HF specialist review in settings where imaging is delayed. External and prospective validation is required before clinical implementation.

## Data Availability

The original contributions presented in the study are included in the article/Supplementary Material, further inquiries can be directed to the corresponding author/s.
