# Supplementary material for: Machine learning-based identification of heart failure with reduced ejection fraction using routine laboratory indicators
Source: Front Cardiovasc Med. 2026 Aug 10;13:1856570. doi: 10.3389/fcvm.2026.1856570 (PMC13500526; doi:10.3389/fcvm.2026.1856570)
Supplement: Supplementary file 1 [file Datasheet1.docx]

**Supplementary material**

Supplementary Tables S1-S5 and Supplementary Figures S1-S2 provide threshold analysis, benchmark reclassification metrics, final hyperparameters, phenotype-sensitivity analysis, SHAP feature importance, subgroup analyses, and logistic-regression odds ratios.

Supplementary Table S1. Threshold-specific sensitivity and negative predictive value of the random forest model for prioritized HFrEF triage.

Sensitivity and negative predictive value (NPV) were calculated for the random forest (RF) model at different classification thresholds. Lower thresholds favored higher sensitivity and NPV, which is appropriate for a rule-out triage strategy aimed at prioritizing echocardiography for suspected HFrEF. RF, random forest; HFrEF, heart failure with reduced ejection fraction; NPV, negative predictive value.

| **Model** | **Threshold** | **Sensitivity** | **NPV** |
| --- | --- | --- | --- |
| RF | 0.1 | 0.938053097 | 0.941666667 |
| RF | 0.15 | 0.911504425 | 0.934640523 |
| RF | 0.2 | 0.89380531 | 0.932960894 |
| RF | 0.25 | 0.814159292 | 0.905405405 |

Supplementary Table S2. Incremental value of the 13-variable random forest model against benchmark models.

The multimarker RF model was compared with a proBNP-only benchmark and a clinical baseline model comprising proBNP, age, sex, and BUN. NRI, net reclassification improvement; IDI, integrated discrimination improvement.

| **Comparison** | **Old_AUC** | **New_AUC** | **NRI** | **IDI** |
| --- | --- | --- | --- | --- |
| RF(13 vars) vs proBNP | 0.766903724 | 0.789428655 | 0.5346 | 0.0873 |
| RF(13 vars) vs Clinics | 0.743095474 | 0.789428655 | 0.4332 | 0.0750 |

Supplementary Table S3. Final optimized hyperparameters for the machine-learning models.

Blank cells indicate parameters that were not applicable to the corresponding algorithm.

| **Model** | **classifier__C** | **classifier__max_depth** | **classifier__n_estimators** | **classifier__learning_rate** | **classifier__n_neighbors** | **classifier__hidden_layer_sizes** |
| --- | --- | --- | --- | --- | --- | --- |
| LR | 0.1 |  |  |  |  |  |
| RF |  | 10 | 100 |  |  |  |
| XGB |  |  |  | 0.01 |  |  |
| SVM | 1 |  |  |  |  |  |
| KNN |  |  |  |  | 5 |  |
| MLP |  |  |  |  |  | (64, 32) |

Supplementary Table S4. Phenotype-sensitivity analysis for HFrEF versus HFpEF classification.

AUC was calculated for the HFrEF-versus-HFpEF sensitivity analysis.

| **Task** | **Test_AUC** |
| --- | --- |
| HFrEF vs HFpEF | 0.82903204 |

Supplementary Table S5. SHAP feature importance of the final random forest model.

Features are ranked by mean absolute SHAP value. SHAP, SHapley Additive exPlanations.

| **index** | **Mean_SHAP** |
| --- | --- |
| proBNP | 0.111201964 |
| TBil | 0.017563529 |
| HCT | 0.030582009 |
| BUN | 0.015397412 |
| B2_MG | 0.015489305 |
| CRP | 0.011646478 |
| DBil | 0.023807947 |
| UA | 0.018581113 |
| Glb | 0.019791026 |
| GGT | 0.017975615 |
| MCH | 0.011348506 |
| LDL_C | 0.017469735 |
| FIB | 0.008607731 |

Supplementary Figure S1. Subgroup analysis of random forest model discrimination.

The forest plot summarizes test-set AUCs across prespecified strata, including age and BUN subgroups, to evaluate potential confounding and performance stability.

Supplementary Figure S2. Standardized logistic-regression odds ratios.

Odds ratios from the standardized LR model provide a transparent benchmark interpretation complementary to SHAP analysis.
